# Androgen-regulation of the protein tyrosine phosphatase PTPRR activates ERK1/2 signalling in prostate cancer cells

**DOI:** 10.1186/s12885-015-1012-8

**Published:** 2015-01-16

**Authors:** Jennifer Munkley, Nicholas P Lafferty, Gabriela Kalna, Craig N Robson, Hing Y Leung, Prabhakar Rajan, David J Elliott

**Affiliations:** 1 Institute of Genetic Medicine, Newcastle University, Newcastle-upon-Tyne, NE1 3BZ UK; 2 Cancer Research UK Beatson Institute, Glasgow, G61 1BD UK; 3 Institute of Cancer Sciences, University of Glasgow, Glasgow, G12 8QQ UK; 4 Northern Institute for Cancer Research, Newcastle University, Newcastle-upon-Tyne, NE2 4HH UK

**Keywords:** PTPRR, RAS/ERK1/2, MAP Kinase, Androgens, Prostate cancer

## Abstract

**Background:**

Androgens drive the onset and progression of prostate cancer (PCa) via androgen receptor (AR) signalling. The principal treatment for PCa is androgen deprivation therapy, although the majority of patients eventually develop a lethal castrate-resistant form of the disease, where despite low serum testosterone levels AR signalling persists. Advanced PCa often has hyper-activated RAS/ERK1/2 signalling thought to be due to loss of function of key negative regulators of the pathway, the details of which are not fully understood.

**Methods:**

We recently carried out a genome-wide study and identified a subset of 226 novel androgen-regulated genes (PLOS ONE 6:e29088, 2011). In this study we have meta-analysed this dataset with genes and pathways frequently mutated in PCa to identify androgen-responsive regulators of the RAS/ERK1/2 pathway.

**Results:**

We find the *PTGER4* and *TSPYL2* genes are up-regulated by androgen stimulation and the *ADCY1, OPKR1, TRIB1, SPRY1* and *PTPRR* are down-regulated by androgens. Further characterisation of PTPRR protein in LNCaP cells revealed it is an early and direct target of the androgen receptor which negatively regulates the RAS/ERK1/2 pathway and reduces cell proliferation in response to androgens.

**Conclusion:**

Our data suggest that loss of PTPRR in clinical PCa is one factor that might contribute to activation of the RAS/ERK1/2 pathway.

**Electronic supplementary material:**

The online version of this article (doi:10.1186/s12885-015-1012-8) contains supplementary material, which is available to authorized users.

## Background

Prostate cancer (PCa) is the most commonly-diagnosed malignancy in men [1], and is driven by androgen hormones acting via their cognate nuclear androgen receptor (AR) transcription factor. The AR exerts its transcriptional effects by binding to DNA sequences termed androgen response elements (AREs) within promoter regions of a number of androgen-regulated genes, including genes encoding cell cycle regulators and regulators of central metabolism and biosynthesis [2]. An important feature of PCa is prognostic heterogeneity: while some prostate cancers can remain indolent for many years others can become much more rapidly aggressive. Distinguishing key signatures between these different cancer types is a key goal. Androgen deprivation therapy (ADT) is the principal treatment for advanced PCa, although, over time, the disease becomes castration-resistant (CRPCa) with limited treatment options [3]. Persistence of AR signalling and reprogramming of the AR transcriptional landscape may underlie progression to CRPCa [4,5], and highlights the importance of AR biology in advanced PCa. Hence, increasing our understanding of the AR signalling in PCa cells should lead to more effective treatment strategies for advanced PCa.

Recently, reciprocal cross-talk between the PI3K pathway and AR signalling has been highlighted as a potential mechanism underlying CRPCa [6]. Alterations in PI3K signalling in advanced PCa are predominantly driven by loss of the tumour suppressor gene *PTEN* which contributes to the progression to invasive disease [7-9]. Another common feature of advanced PCa is hyperactivation of the RAS/ERK1/2 pathway [10-12] thought to be driven by loss of function of key negative regulators of the pathway [13]. Although RAS/ERK1/2 activation alone cannot initiate PCa development, it can serve as a potentiating second hit to loss of PTEN to accelerate PCa progression [13].

Because of its established importance in clinical prostate cancer, the identification of new mechanisms through which the RAS/RAF/MAPK/ERK pathway is regulated is of great interest. We recently carried out genome-wide exon-specific profiling of PCa cells to identify novel androgen-regulated transcriptional events [14]. As well as identifying a number of alternative mRNA isoforms [15], we also identified a subset of 226 novel androgen-regulated genes [14]. In the light of evidence implicating cross-talk with AR, we searched this dataset for novel androgen-regulated genes associated with RAS/ERK1/2 signalling.

## Methods

### IPA Pathway analysis

Gene lists from Rajan *et al.* [14] were uploaded to the web-based Ingenuity Pathway Analysis (IPA; Ingenuity Systems) software programme, and the “Core Analysis” function was used to study direct and indirect regulatory relationships between genes and their known biological functions.

### Antibodies

The following antibodies were used in the study: anti-PTPRR rabbit polyclonal (17937 Proteintech), anti- phospho-p44/42 MAPK mouse monoclonal (Erk1/2 Thr202/Tyr204) (Cell signalling 9106), anti-ERK2 mouse monoclonal (1647 Santa Cruz), anti-actin rabbit polyclonal (A2668, Sigma), anti-AR mouse antibody (BD Bioscience, 554226), anti-FLAG mouse monoclonal (F3165, Sigma), anti-PTEN rabbit polyclonal (Cell Signalling 138G6), anti-α-Tubulin mouse monoclonal (Sigma T5168), normal rabbit IgG (711-035-152 Jackson labs) and normal mouse IgG (715-036-150 Jackson labs). The specificity of the PTPRR antibody was confirmed by blocking with the immunising peptide (ag12145 Proteintech) (Additional file 1: Figure S2).

### esiRNA

esiRNAs PTPRR and AR were obtained from Sigma-Aldrich (EHU078991 and EHU025951).

### DNA constructs

*PTPRR* cloned into pCDNA3.1 was a kind gift from Mirco Menigatti, University of Zurich. The *PTPRR* open reading frame was subsequently cloned into pCDNA5 using *Not*I and *Xho*I for creation of the Flp-In™-293 stable cell line.

### Cell culture

Cell culture and androgen treatment of cells was as described previously [14,15]. All cells were grown at 37°C in 5% CO_2_. LNCaP cells (CRL-1740, ATCC) were maintained in RPMI-1640 with L-Glutamine (PAA Laboratories, R15-802) supplemented with 10% Fetal Bovine Serum (FBS) (PAA Laboratories, A15-101). For androgen treatment of LNCaP cells, medium was supplemented with 10% dextran charcoal stripped FBS (PAA Laboratories, A15-119) to produce a steroid-deplete medium. Following culture for 72 hours, 10 nM synthetic androgen analogue methyltrienolone (R1881) (Perkin–Elmer, NLP005005MG) was added (Androgen +) or absent (Steroid deplete) for the times indicated. Where indicated, LNCaP cells were pre-treated for 1 hour with vehicle (dimethylsulfoxide; DMSO) (Sigma, C1988) or 1 μg/ml cycloheximide (Sigma, D2438) prior to addition of 10 nM R1881 for 24 hours as previously described [16]. Similarly, LNCaP cells were pre-treated with with 10 μM bicalutamide (Casodex, AstraZeneca) or ethanol (vehicle) for 2 hours prior to addition of 10 nM R1881 for 24 hours.

PC-3 (CRL-1435, ATCC), PC-3 M [17], CWR22Rv1 (CRL-2505, ATCC), DU145 (HTB-81, ATCC), and BPH-1 cells [18] were maintained in RPMI-1640 with L-Glutamine supplemented with 10% FBS. LNCaP-AI and LNCaP-cdxR were derived from LNCaP parental cells and maintained as previously described [19,20].

Stable LNCaP cell lines were generated by transfecting cells using Lipofectamine 2000 (11668-027, Invitrogen), followed by selection with 300 μg/ml Geneticin (Invitrogen, 10131019) (reduced to 150 μg/ml following the death of untransfected cells) for at least four weeks. Flp-In™-293 cells (R750-07, Invitrogen) were maintained in DMEM GlutaMax (Invitrogen, 10566-040), supplemented with 10% FBS (PAA Laboratories, A15-101) and stable cell lines generated using the Flp-In T-Rex Core Kit (K6500-01, Invitrogen) according to the manufacturer’s instructions. Protein expression was induced using 1 μg/ml tetracycline (T7660, Sigma) for 72 hours.

### RT-qPCR

Cells were harvested and total RNA extracted using TRIzol (Invitrogen, 15596-026), according to manufacturer’s instructions. RNA was treated with DNase 1 (Ambion) and cDNA was generated by reverse transcription of 1 μg of total RNA using the Superscript VILO cDNA synthesis kit (Invitrogen, 11754-050). Quantitative PCR (qPCR) was performed in triplicate on cDNA using SYBR® Green PCR Master Mix (Invitrogen, 4309155) using Applied Biosystems 7900HT. Samples were normalised using the average of three reference genes: *GAPDH*, β –tubulin and *actin*. All primer sequences are listed in Additional file 2: Table S2.

### Proliferation assay

EdU incorporation was measured over 6 hours using the Click-iT® EdU Alexa Fluor® 488 Imaging Kit (Invitrogen, C10337) and counted using ImageJ. At least 3000 cells were counted for each cell line across 3 coverslips. MTT cell proliferation assay was carried out as per the manufacturer’s instructions (Cayman, 10009365) starting with 20,000 cells per well, with 9 replicates per sample.

### Clinical samples

Six protein lysates from primary clinical prostate tumours were used in this study. Full ethical approval was obtained for human sample collection from the Northumberland, Tyne and Wear NHS Strategic Health Authority Local Research Ethics Committee (Ref: 2003/11) and written informed consent for the use of surgically obtained tissue was provided by all patients.

## Results

### Genes encoding components of RAS/ERK1/2 signalling pathways are regulated by androgens in PCa cells

**Figure 1:**
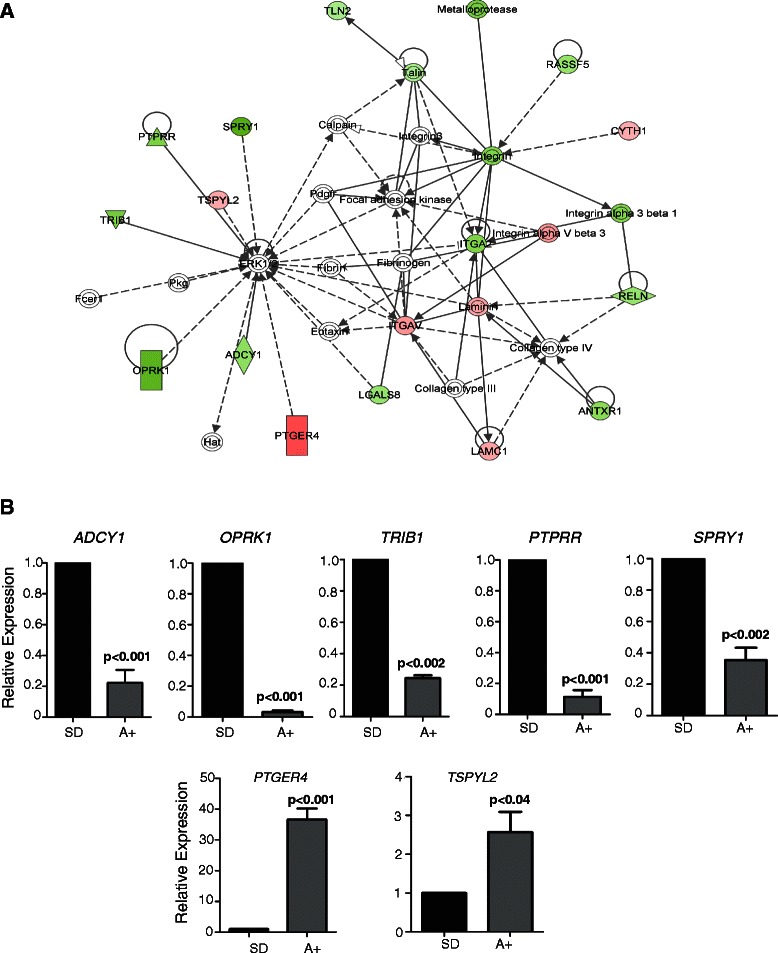
**IPA Pathway Analysis of androgen regulated RAS/ERK1/2 signalling.** Gene expression changes in LNCaP cells cultured in the presence or absence of androgens for 24 hours. **(A)** Gene lists from Rajan et al. [14] were uploaded to the web-based Ingenuity Pathway Analysis (IPA; Ingenuity Systems) software programme, and used to identify novel androgen-regulated genes associated with RAS/ERK1/2 signalling. Genes up-regulated by androgens are highlighted in red, and down-regulated genes are in green. Further details are shown in Additional file 1: Table S1. This highlighted the already well-characterised *SPRY1* as an androgen-regulated negative regulator of ERK1/2, as well as some additional previously uncharacterised targets, *PTGER4*, *TSPYL2*, *ADCY1*, *OPKR1*, *TRIB1*, *PTGER4* and *PTPRR*. **(B)** Expression changes for 7 of these genes were validated by real-time PCR.

Complete gene lists from our ExonArray dataset [14] were manually curated for androgen-regulated changes within genes associated with RAS/ERK1/2 signalling. We identified potent down-regulation of *SPRY1* expression in response to androgens (Log_2_FC = -2.37 p < 0.001). Full gene lists were then uploaded to the web-based Ingenuity Pathway Analysis (IPA; Ingenuity Systems) software programme, and the IPA ‘Core Analysis’ function was used to identify novel androgen-regulated genes within pathways associated with *SPRY1* (Figure 1A & Additional file 3: Table S1). This network analysis identified a number of novel androgen-regulated genes previously linked to the RAS/RAF/MAPK/ERK signalling pathway. We confirmed androgen regulation of these genes in LNCaP cells using real-time PCR (Figure 1B). Two genes, *PTGER4* and *TSPYL2* were up-regulated in response to androgens, whereas five others, *ADCY1*, *OPKR1*, *TRIB1*, *SPRY1* and *PTPRR* were repressed.

### PTPRR is an early and direct target of the AR at the mRNA and protein level

**Figure 2:**
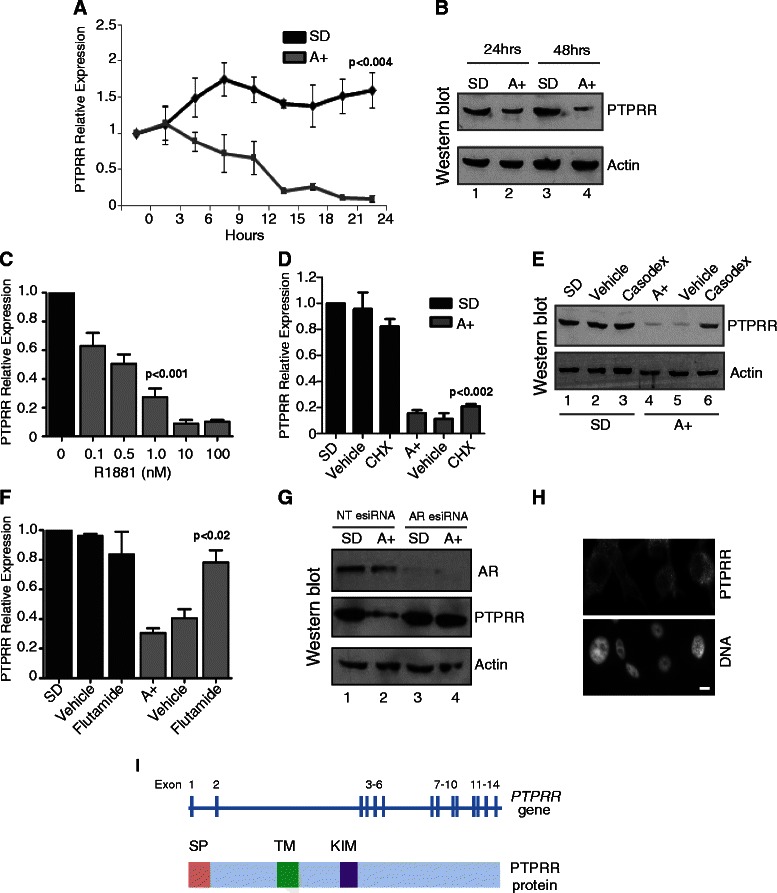
***PTPRR*expression is an early and directly repressed target of the AR. (A)** LNCaP cells were cultured in medium supplemented with 10% dextran charcoal stripped FBS to produce a steroid deplete medium. Following culture for 72 hours, cells were treated with 10 nM synthetic androgen analogue methyltrienolone (R1881) for the times indicated. **(A)** Expression of *PTPRR* mRNA in cells grown in steroid deplete (SD) or androgen (A+) treated conditions over a 24 hours period. The response to androgens was confirmed using PSA (KLK3) expression (not shown). **(B)** Expression of PTPRR protein is reduced in LNCaP cells treated with 10 nM R1881 for 24 and 48 hours as detected by western blot. Actin was used as a loading control. **(C)** Repression of PTPRR is also evident in LNCaP cells treated with 0.1 to 100 nM of R1881. Relative *PTPRR* expression was detected by real-time PCR. **(D)** The reduction in *PTPRR* mRNA expression in response to androgens is still seen in the presence of 1 μg/ml cycloheximide (CHX) as confirmed by real-time PCR. **(E)** Repression of PTPRR protein expression by the AR is inhibited in the presence of 10 μM of the anti-androgen Casodex (bicalutamide) (lane 6) and by 10 μM of flutamide **(F)**. **(G)** Depletion of AR protein in LNCaP cells by esiRNA shows that when the AR is depleted there is no reduction in PTPRR protein in response to androgens. **(H)** Immunofluorescent staining of LNCaP cells grown in steroid depleted conditions indicates that PTPRR is localised to the cytoplasm. Bar is 10 μM. The specificity of the antisera was confirmed with pre-absorption with the immunising peptide, and by detection of over-expressed protein and esiRNA mediated protein depletion (Additional file 3: Figure S2, Figure 3A and 3D). **(I)** The PTPRR gene and protein structure is illustrated. The gene consists of 14 exons, and codes for a 74 kDa protein which contains a signal peptide (SD domain), a transmembrane domain (TM) and a kinase interaction motif (KIM).

**Figure 3:**
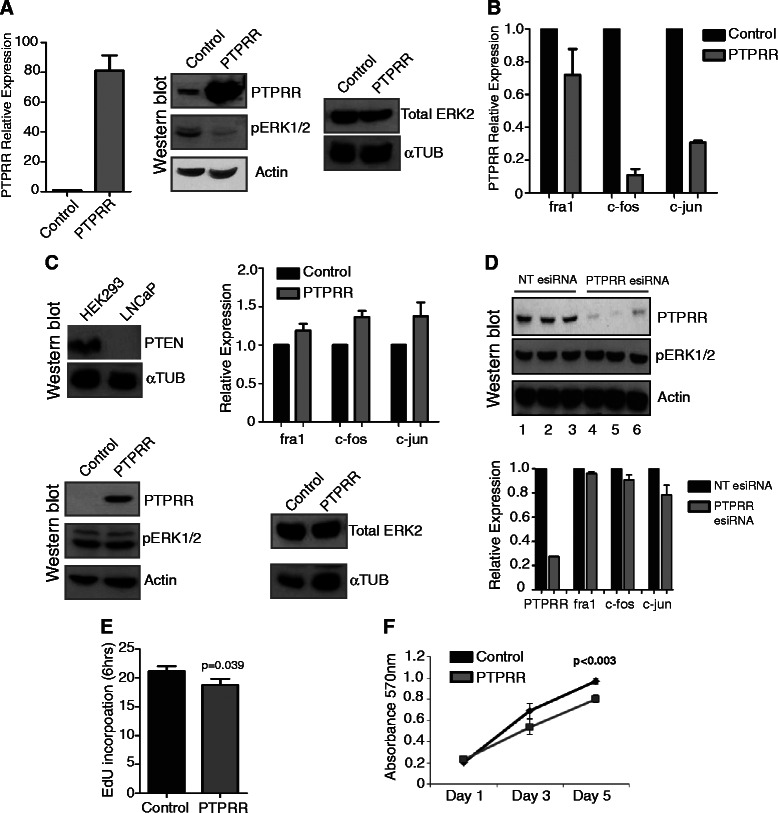
**Expression of*PTPRR*in androgen treated LNCaP cells reduces ERK1/2 signalling and decreases cell proliferation in the presence of androgens. (A)** Over-expression of PTPRR in our LNCaP stable cell lines was confirmed by real-time PCR and by western blotting (**A**, left and middle panels). Analysis by western blot revealed that cells over-expressing PTPRR had decreased levels of phospho-ERK1/2 (pERK1/2). Actin was used as a loading control (**A**, middle panels). Total levels of ERK2 did not change in these cells (**A**, right panel). **(B)** Analysis of three transcription factors: *fra1*, *c-fos* and *c-jun*, in our stable LNCaP cells by real-time PCR in the presence of androgens revealed decreased expression levels for all three targets in cells over-expressing PTPRR. *fra1*, *c-fos* and *c-jun* are downstream targets of pERK1/2. **(C)** Western blotting confirmed our LNCaP cells to be PTEN negative and our HEK293 cells to be PTEN positive (**C**, upper left panels). In contrast to what was seen for LNCaP cells, there is no change in pERK1/2 levels in HEK293 cells over-expressing PTPRR. There was no change in total ERK2 levels. ɑ-tubulin was used as a loading control (**C**, lower left and lower right panels). There was also no change in the expression of *fra1*, *c-fos* or *c-jun* expression as detected by real-time PCR (**C**, upper right panel). **(D)** esiRNA mediated depletion of PTPRR in steroid depleted LNCaP cells. Despite loss of PTPRR, there is no effect on the levels of pERK1/2 (upper panel lanes 3-6) or on the expression of *fra1*, *c-fos* or *c-jun* (lower panel, real-time PCR). **(E)** LNCaP cells over-expressing PTPRR and grown in the presence of androgens, had a lower percentage of cells in S phase, as detected by EdU incorporation (p = 0.039), and also had an impaired rate of proliferation as measured by MTT assay (p < 0.003) **(F)**.

The above network analysis suggested that the *PTPRR* gene is a novel androgen regulated target in the MAPK/ERK signalling network. The genomic loci of AR binding sites mapped by ChIP in LNCaP cells [4] were uploaded onto the UCSC genome browser. Three known AR binding sites were identified in the vicinity of the *PTPRR* gene, one of which was less than 5 kb upstream, and another within an internal intronic region (Additional file 4: Figure S1). To test whether the *PTPRR* gene might be under direct control of androgens through AR regulation, we examined *PTPRR* expression in LNCaP cells grown in steroid depleted medium and in cells treated with 10 nM of the synthetic androgen analogue R1881 (methytrienolone) by real-time qPCR over a 24 hour period (Figure 2A), and at the protein level over 48 hours by western blotting (Figure 2B). The specificity of the PTPRR antibody used was confirmed by peptide blocking, detection of over-expressed protein and detection of esiRNA mediated protein depletion (Figure 2B, Figure 3A and D and Additional file 1: Figure S2). *PTPRR* expression was rapidly reduced by 10 nM R1881 treatment at both the mRNA and protein level. Repression of the *PTPRR* gene and protein was also observed with a range of R1881 concentrations from 0.1 nM to 100 nM (Figure 2C). To test whether androgen-mediated suppression of *PTPRR* expression was a direct result of AR activity, we treated LNCaP cells with 10 nM R1881 in the presence and absence of cycloheximide to inhibit de novo protein synthesis. Androgen-mediated down-regulation of *PTPRR* mRNA expression was still observed in the presence of the protein synthesis inhibitor cycloheximide indicating that PTPRR repression might be directly mediated by the AR (Figure 2D). Confirming this, we found androgen-mediated *PTPRR* protein reduction was prevented by the AR antagonists casodex (Figure 2E), and flutamide (Figure 2F), and when cells are depleted of AR using esiRNA (Figure 2G). Immunofluorescent staining of LNCaP cells grown in the absence of androgens indicates that PTPRR protein localises to the cytoplasm (Figure 2H). The structure of the *PTPRR* gene and protein are illustrated in Figure 2I.

### Re-expression of PTPRR in androgen treated LNCaP cells reduces phosphorylation of ERK1/2 and regulates downstream oncogenic transcription factors

The above data predicted that AR-regulated *PTPRR* suppression in PCa cells may contribute to modulation of RAS/ERK signaling in response to androgens. To test this prediction, we created a stable LNCaP cell line in which *PTPRR* was expressed under the control of the CMV promoter and a control stable cell line transfected with empty vector. This CMV promoter is active independent of androgen stimulation. In the stable cell line made with *PTPRR* , increased PTPRR gene expression was detected at both the RNA level (by qRT-PCR relative to three housekeeping genes) and protein level (by western analysis, relative to actin) compared to the control cell line made with empty vector (Figure 3A left and middle panels).

Consistent with stable expression of PTPRR being sufficient to dampen activity of the MAPK/ERK network, LNCaP cells over-expressing PTPRR protein also showed reduction in phosphorylated ERK1/2 in the presence of androgens (Figure 3A middle panel). As a parallel loading control, no change in total ERK1/2 levels was detected (Figure 3A right panel). Correlating with this modulation of the RAS/ERK pathway in response to PTPRR expression, we also observed parallel repression of three oncogenic transcription factors (*c-fos*, *fra1* and *c-jun*) known to be downstream regulated targets of ERK1/2 [21] (Figure 3B).

Prostate cancer cells can be genetically heterogeneous, and this can have important implications for prognosis and treatment. We recently found a synergistic effect between loss of *Pten* and *Spry2* in murine PCa progression [22]. To test if the ability of PTPRR to negatively regulate MAPK signalling might depend on cellular background we generated a second stable cell line over-expressing PTPRR in the HEK293 cell background which are not derived from PCa cells but are PTEN positive (Figure 3C top left panel). Consistent with an important role for cell background in the response to PTPRR expression, over-expression of PTPRR did not reduce either phosphorylation of ERK1/2 or the expression of *c-fos*, *fra1* or *c-jun* in the HEK293 cell background (Figure 3C). To test whether endogenous levels of PTPRR alone were sufficient to repress phosphorylation of ERK in the absence of other signals in androgen-treated LNCaP cells, we depleted PTPRR from androgen-depleted cells. Even though over-expression of PTPRR in androgen treated LNCaP cells was sufficient to repress ERK1/2 phosphorylation, depletion of PTPRR alone by esiRNA in steroid depleted LNCaP cells was insufficient to restore ERK1/2 phosphorylation (Figure 3D). This result suggests that although PTPRR has an important role in this pathway, additional androgen-regulated proteins are involved in modulating ERK1/2 phosphorylation in response to androgens. These additional genes likely include other members of the RAS/ERK1/2 pathway (identified by our IPA pathway analysis in Figure 1).

### Over-expression of PTPRR reduces LNCaP cell proliferation

The down-regulation of *c-fos*, *fra1* and and *c-jun* expression in stable LNCaP cells over-expressing PTPRR suggested that androgen-mediated down-regulation of *PTPRR* expression might be important for proliferation of LNCaP cells. To test for such an effect on cell proliferation, we measured the proportion of cells in S-phase in our stable LNCaP cell line expressing *PTPRR*, and control LNCaP cells, using incorporation of EdU. Consistent with a reduced rate of proliferation, LNCaP cells over-expressing *PTPRR* in the presence of androgens had a reduced amount of cells in S phase over a six hour period (from 21.2% to 18.8%) relative to control cells (p < 0.04) (Figure 3E). A decrease in LNCaP cell growth in response to PTPRR expression was confirmed using an MTT assay, where there was a significant reduction in cell proliferation in *PTPRR*-expressing cells (p < 0.003) (Figure 3F).

### Low expression of PTPRR in Invasive PCa cell lines and Clinical PCa tissue

**Figure 4:**
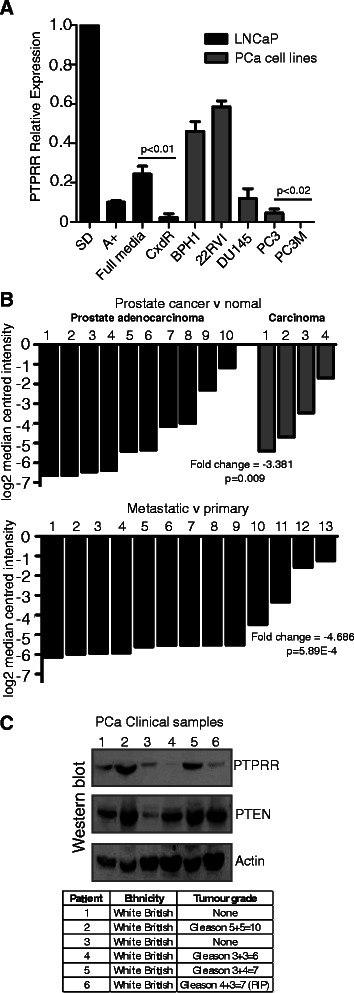
***PTPRR*expression in prostate cancer cell lines and in clinical PCa tissue. (A)** Real time PCR analysis of PTPRR expression in 7 prostate cancer cell lines. The LNCaP cell lines used were: androgen responsive LNCaP cells grown in the presence (A+) or absence (SD) of androgens (as described in Figure 1), and in the presence of 10% FBS (full media, FM), androgen insensitive LNCaP cells (AI) grown in charcoal stripped FBS, and Casodex resistant LNCaP cells grown in the presence of 10 μM of the anti-androgen Casodex. The additional cell lines were: benign prostate hyperplasia cells (BPH1), androgen independent CWR22RVI, AR-/ PTEN+ DU145 cells, AR+/PTEN- PC3 cells and their metastatic derivative PC3M. All additional cell lines were grown in 10% FBS. For further details of the cell lines used see Methods. For real-time PCR samples were normalised to 3 housekeeping genes (actin, GAPDH and β-tubulin), and relative to the expression of *PTPRR* in steroid depleted LNCaP cells. **(B)** Expression of *PTPRR* mRNA in prostate cancer clinical samples as measured by Ramaswamy et al. [23,24]. The upper panel shows fold change in 14 prostate cancer tissue samples relative to normal prostate tissue, and the lower panel shows fold change in 13 metastatic samples relative to primary prostate cancer tissue, both measured by Affymetrix Array. **(C)** Analysis of PTPRR and PTEN protein expression in 6 clinical prostate samples by western blotting. Actin was used as a loading control. The tumour grade and the patient ethnicity for each sample are shown in the table.

We next examined *PTPRR* mRNA expression in a panel of PCa cell lines of different invasive capabilities, and with differing expression of PTEN (Figure 4A). Consistent with AR-dependent down-regulation, there was reduced *PTPRR* expression in the casodex resistant LNCaP derivative cell line relative to the androgen sensitive LNCaP cells (p < 0.003) (Figure 4A). Consistent with potential changes in *PTPRR* expression over disease progression, in these cell line models there was a low level of expression of *PTPRR* in PC3 cells, but expression was undetectable in their metastatic derivative PC3M.

The above data show that decreased *PTPRR* expression can be important to prostate cancer cell proliferation through modulation of the MAPK/ERK pathway, and this might be modulated dependent on cellular background including PTEN status. We examined *PTPRR* gene expression in prostate cancer clinical samples using previously published datasets that are publically available. Comparison of *PTPRR* expression by Affymetrix Array of 14 samples reported a 3.381 fold reduction in prostate cancer relative to normal tissue [23], and a 4.686 fold reduction in metastatic versus primary prostate cancer [24] (Figure 4B). *PTPRR* expression was also significantly reduced in 3 additional previously published datasets [25-27] with 2 other datasets showing no significant changes in *PTPRR* gene expression [11,28].

We tested PTPRR protein expression in a small panel of clinical PCa samples. Although this was a small sample set, the data suggested PTPRR has a heterogeneous expression profile in prostate tumour clinical samples. PTPRR expression was detected in half the samples but either low or undetectable in 3/6 samples (samples 3,4 and 6) despite high expression of four other control proteins (not shown). PTEN expression was also detected in 5/6 of these clinical PCa samples, but one tumour (sample 3) had low levels of both PTPRR and PTEN. Although this sample of tumours is small, these data suggest individual PCa patients have heterogeneous patterns of PTPRR expression relative to other potential modifier genes (Figure 4C, western blot with patient information shown below), and might contribute to the known heterogeneity of prostate tumours.

## Discussion

A common feature of aggressive PCa is hyperactivation of the RAS/ERK1/2 pathway [10,11]. ERK1/2 signalling is known to play an important role in PCa development [29] and activation of ERK1/2 has been correlated with malignancy [10-12]. One possible mechanism for RAS/ERK1/2 hyper-activation is loss of function of key negative regulators of the pathway, such as the *Sprouty* genes. *SPRY1* and *SPRY2* function primarily as physiological negative regulators of the RAS/ERK1/2 pathway and act to suppress prostate tumourigenesis [13,30]. *SPRY1* and *SPRY2* are commonly inactivated in PCa where they are linked to disease progression [22,31,32]. Using network analysis and a meta-analysis of prior exon array data, we identify concomitant down-regulation of a novel androgen-regulated gene, phosphatase receptor type R (*PTPRR*) as well as the known RAS/ERK1/2 pathway negative regulator *SPRY1*.

Here we show data to suggest that *PTPRR* is a direct AR target gene, and is rapidly repressed by androgens in LNCaP cells. We also demonstrate that over-expression of *PTPRR* in androgen stimulated cells is sufficient to decrease phosphorylation of ERK1/2 and reduce both the expression of oncogenic transcription factors and proliferation of prostate cancer cells. The *PTPRR* gene encodes a classical transmembrane protein-tyrosine phosphatase (PTP) receptor type R (PTPRR). PTPRR is normally expressed in the brain, placenta, small intestine, stomach, uterus and weakly in the prostate [33]. Mouse gene *Ptprr* encodes multiple protein tyrosine phosphatase receptor type R (PTPRR) isoforms, which display distinct patterns of expression during neural development [34,35], and negatively regulate mitogen-activated protein kinase (MAPK) signalling pathways; both ERK1 and ERK2 are hyperphosphorylated in the brains of mice deficient for PTPRR [36]. In cell lines PTPRR has been shown to de-phosphorylate p44/42 ERK1/2 in response to growth factors [37,38]. Repression of PTPRR expression via methylation has been detected in pre-cancerous colorectal lesions and in cervical adenocarcinoma [39,40], and in cervical cancer PTPRR may have a role in metastasis and be a biomarker of invasive cervical cancer [21]. PTPRR expression has also recently been identified as a prognostic indicator in oral squamous cell carcinoma [41].

Prostate tumours show huge biological heterogeneity, with some patients living for 20 years with organ confined disease, while others progress to lethal metastatic disease within 2 years of diagnosis. A deeper understanding of this genomic diversity will help identify genomic changes which can help distinguish indolent from aggressive PCa. The RAS/ERK1/2 signalling pathway is mutated in 43% of primary PCa tumours and 90% of PCa metastases [11]. Although *PTPRR* is only mutated in 1% of PCa tumours [11], our data demonstrates that it is a key component of the clinically important RAS/ERK1/2 signalling pathway, and that its expression level can have a clear affect on the activity of this pathway. Recent work has shown that although RAS/ERK1/2 activation alone cannot initiate PCa development, RAS/ERK1/2 and PTEN loss cooperate to promote EMT and metastasis initiated from PCa progenitor cells [12].

Our data also indicate that the importance of PTPRR expression on the ERK1/2 pathway activity is cell line and context dependent, suggesting cell background is important. One potential source of this cell background might be PTEN. We recently found a synergistic effect in murine PCa progression between loss of PTEN and SPRY2 [22]. We speculate that similar to SPRY2 the effects of PTPRR on ERK1/2 signalling might only be exerted in clinical prostate cancer in the absence of PTEN (e.g. patient 3 in Figure 4C). Further *in vitro* and *in vivo* studies are required to determine whether loss of PTPRR is a consistent event in PCa, and whether reintroduction of PTPRR expression may limit the progression of CRPCa.

## Conclusions

In summary, our study has identified the protein tyrosine phosphatase PTPRR as an early and direct target of the androgen receptor that is rapidly repressed by androgens in prostate cancer cells. Further characterisation of PTPRR protein in LNCaP cells revealed it negatively regulates the RAS/ERK1/2 pathway and reduces cell proliferation in response to androgens. Our data suggest that loss of PTPRR protein might contribute to hyper-activation of the RAS/ERK1/2 pathway and has important implications for the development of therapies targeting this pathway.

## Additional files

Additional file 1: Figure S2. The specificity of the *PTPRR* antibody used was confirmed by peptide blocking with the corresponding immunising peptide.
Additional file 2: Table S2. Primer sequences used.
Additional file 3: Table S1. IPA Pathway analysis scores.
Additional file 4: Figure S1. AR binding sites in ERK1/2 associated genes. Data for AR ChIP-seq peaks was uploaded from Massie et al. [2] supplementary data onto UCSC genome browser custom tracks. The position of AR ChIP-seq peaks detected in LNCaP cells within 100 kb of the PTPRR gene are illustrated (A). Scale bar is 100 kb. The position of AR ChIP-seq peaks detected within 100 kb of *ADCY1*, *OPKR1*, *TRIB1*, *SPRY1, PTGER4* and *TSPYL2* are shown below (B).
