# Supplementary figures and images for: Androgen-regulation of the protein tyrosine phosphatase PTPRR activates ERK1/2 signalling in prostate cancer cells
Source: BMC Cancer. 2015 Jan 16;15:9. doi: 10.1186/s12885-015-1012-8 (PMC4302442; doi:10.1186/s12885-015-1012-8)

Additional file 3: Figure S2

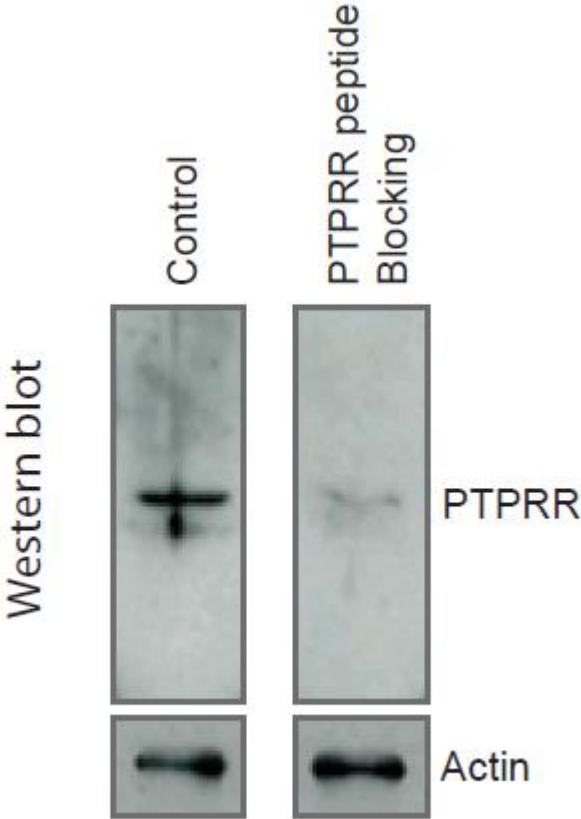

Supplement: Additional file 1: Figure S2. — The specificity of the PTPRR antibody used was confirmed by peptide blocking with the corresponding immunising peptide. [file 12885_2015_1012_MOESM1_ESM.pdf]
