# Supplementary material for: Androgen-regulation of the protein tyrosine phosphatase PTPRR activates ERK1/2 signalling in prostate cancer cells
Source: BMC Cancer. 2015 Jan 16;15:9. doi: 10.1186/s12885-015-1012-8 (PMC4302442; doi:10.1186/s12885-015-1012-8)
Supplement: Additional file 4: Figure S1. — AR binding sites in ERK1/2 associated genes. Data for AR ChIP-seq peaks was uploaded from Massie et al. [2] supplementary data onto UCSC genome browser custom tracks. The position of AR ChIP-seq peaks detected in LNCaP cells within 100 kb of the PTPRR gene are illustrated (A). Scale bar is 100 kb. The position of AR ChIP-seq peaks detected within 100 kb of ADCY1, OPKR1, TRIB1, SPRY1, PTGER4 and TSPYL2 are shown below (B). [file 12885_2015_1012_MOESM4_ESM.pdf]

Additional file 2: Figure S1

A

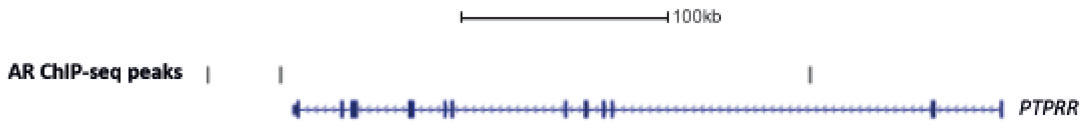

B

	Gene location	AR ChIP-seq peaks	Distance to gene (bp)
PTPRR	chr12: 71031853-7131458	chr12: 70958876-70959445	72,408
		ch12: 71026249-71026985	4,868
		ch12: 71237995-71238552	Within gene (intronic)
ADCY1	chr7: 45614125-45762714	chr7: 45697190-45698375	Within gene (intronic)
OPRK1	chr8: 54138276-54164257		
TRIB1	chr8: 126442563-126450647	chr8: 126426832-126427204	15,359
		chr8: 126515354-126516254	73,691
SPRY1	chr4: 124317950-124324915	chr4: 124405934-124406471	81,556
PTGER4	chr5: 40680032- 40693837	chr5: 40685713-40686082	Within gene (intronic)
		chr5: 40720992-40721463	27,155
		chr5: 40721903-40722381	28,066
TSPYL2	chrX: 53111542-53117728	chrX: 53117533-53117984	195
		chrX: 53132418-53132872	14,690
